# Complete Genome Sequence of Lactobacillus reuteri PNW1, a Promising Probiotic Candidate

**DOI:** 10.1128/MRA.00034-19

**Published:** 2019-02-21

**Authors:** Kazeem A. Alayande, Olayinka A. Aiyegoro, Collins N. Ateba

**Affiliations:** a Department of Microbiology, North-West University, Mmabatho, South Africa; b Unit for Environmental Sciences and Management, North-West University, Potchefstroom, South Africa; c Agricultural Research Council, Animal Production Institute, Gastrointestinal Microbiology and Biotechnology Division, Irene, South Africa; Queens College

## Abstract

This study reports the whole-genome sequence of Lactobacillus reuteri PNW1 isolated from gastrointestinal tracts of weaned piglets of the indigenous South African Windsnyer pig breed. A total of 5.2 GB data comprising 8,209,104 paired-end reads were generated. The assembled genome is 2,430,215 bp long in 420 contigs with 39% G+C content.

## Abstract (precis)

This study reports the whole-genome sequence of Lactobacillus reuteri PNW1 isolated from gastrointestinal tracts of weaned piglets of the indigenous South African Windsnyer pig breed. A total of 5.2 GB data comprising 8,209,104 paired-end reads were generated.

## ANNOUNCEMENT

Gastrointestinal tract infections have been identified as an important health disorder ravaging livestock farms and human well-being (1). Manipulation of the intestinal microbiota through the direct feeding of beneficial microorganisms in the form of probiotics may attenuate the enteric health challenge (2). Probiotics have been argued to fall into the class of the most popular bioactive and healthy functional foods (3). Lactic acid bacteria (LAB) are reported to be the bacterial strains most commonly used as probiotics. Among many other direct beneficial effects to the host system, LAB have the ability to inhibit the growth of pathogens, reduce symptoms of lactose intolerance, and enhance the immune system. LAB have also demonstrated anticarcinogenic activity (4, 5). LAB are typically regarded as safe due to their long history of use as probiotics (6); however, every novel probiotic cannot be assumed to share the historical safety of the conventionally used strains (7). Just as occurs in other organisms, probiotics may possess undesirable properties. Thus, proper safety analysis is required (8).

The candidate probiotic bacterial strain was isolated from the gastrointestinal tracts of compassionately sacrificed weaned piglets of the indigenous South African Windsnyer pig breed. The organism was cultured in de Man-Rogosa-Sharpe broth under strict anaerobic conditions and incubated at 37°C for 24 h in an anaerobic jar provided with an AnaeroGen system (Thermofisher, UK).

The bacterial genomic DNA was extracted with a DNA extraction kit (Zymo Research, USA) following the manufacturer’s instructions. The genome was prepared using an Illumina Nextera DNA Flex library prep kit, and the run was performed on an Illumina MiSeq Platform1 system at the Agricultural Research Council, Biotechnology Platform (Pretoria, South Africa). A total of 5.2 GB data comprising 8,209,104 paired-end reads was generated, with a maximum length of 2 × 300 bp. The data were filtered for low-quality reads and adapter regions using Trimmomatic version 0.32 (9). The adapter sequences were clipped using a mismatch value of 2, a palindrome clip threshold of 30, and a simple clip threshold of 15. A draft genome assembly was constructed using SPAdes version 3.7.1 (10). Genome annotation was carried out by using the NCBI Prokaryotic Genome Annotation Pipeline (PGAP) and Rapid Annotations using Subsystems Technology (RAST) (11). The assembled genome is 2,430,215 bp long in 420 contigs (with protein-encoding genes [PEGs]) with final coverage of 1,248×. The *N*_50_ value is 28,048 bp, the number of contigs that are ≥ *N*_50_ is 24, and the average G+C content is 39%. The numbers of protein-coding sequences and structural RNAs are 2,581 and 79, respectively. *In silico* analyses of the genome sequence using Antibiotics and Secondary Metabolites Analysis SHell version 4.2.0 (12) identified seven different secondary metabolites on nine gene clusters. All software was employed with the default parameters. Further analysis is under way on detailed safety evaluation of the isolate.

The procedures involved in this study complied with all relevant legislation regarding the protection of animal welfare and were approved by the Agricultural Research Council, Animal Production Institute Ethics Committee (APIEC13/008).

### Data availability.

This whole-genome shotgun project has been deposited at DDBJ/ENA/GenBank under the accession number RJWE00000000. The version described in this paper is version RJWE01000000. The SRA accession number is PRJNA504734.
